# Living environment, social support, and informal caregiving are associated with healthcare seeking behaviour and adherence to medication treatment: A cross‐sectional population study

**DOI:** 10.1111/hsc.12758

**Published:** 2019-04-23

**Authors:** Erik Berglund, Per Lytsy, Ragnar Westerling

**Affiliations:** ^1^ Department of Public Health and Caring Sciences Uppsala University Uppsala Sweden; ^2^ Division of Insurance Medicine, Department of Clinical Neuroscience Karolinska Institute Stockholm Sweden

**Keywords:** adherence, healthcare utilisation, housing type, informal caregiving, neighbourhood, social support

## Abstract

Despite the well‐known associations between local environment and health, few studies have focused on environment and healthcare utilisation, for instance healthcare seeking behaviour or adherence. This study was aimed at analysing housing type, behaviour based on perceived local outdoor safety, social support, informal caregiving, demographics, socioeconomics, and long‐term illness, and associations with health‐seeking and adherence behaviours at a population level. This study used data from the Swedish National Public Health Survey 2004–2014, an annually repeated, large sample, cross‐sectional, population‐based survey study. In all, questionnaires from 100,433 individuals were returned by post, making the response rate 52.9% (100,433/190,000). Descriptive statistics and multiple logistic regressions were used to investigate associations between explanatory variables and the outcomes of refraining from seeking care and non‐adherence behaviour. Living in rented apartment, lodger, a dorm or other was associated with reporting refraining from seeking care (adjusted OR 1.16, 95% CI 1.00–1.22), and non‐adherence (adjusted OR 1.22; 95% CI 1.13–1.31). Refraining from going out due to a perceived unsafe neighbourhood was associated with refraining from seeking care (adjusted OR 1.59, 95% CI 1.51–1.67) and non‐adherence (adjusted OR 1.26, 95% CI 1.17–1.36). Social support and status as an informal caregiver was associated with higher odds of refraining from seeking medical care and non‐adherence. This study suggests that living in rental housing, refraining from going out due to neighbourhood safety concerns, lack of social support or informal caregiver status are associated with lower health‐seeking behaviour and non‐adherence to prescribed medication.

**What is known on this topic**

- Environmental attributes are associated with health outcomes and some health behaviours.
- The socioeconomic status of an individual is associated with their living environment.
- Lack of social support and being an informal caregiver are risk factors for stress, illness, and poorer health.

**What this study adds**

- Living in rental housing and refraining from going out in the neighbourhood due to safety concerns were associated with refraining from seeking medical care. Not living in a private house and refraining from going out in the neighbourhood due to safety concerns were associated with non‐adherence to prescribed medication.
- Persons providing unpaid care to a sick or disabled family member or friend (being informal caregivers) were more likely than non‐caregivers to report non‐adherence to treatment, and to report refraining from seeking medical care for their own care needs.
- Lack of social support, and having financial problems or a long‐term illness were associated with refraining from seeking medical care and non‐adherence to prescribed medication.

## 1 INTRODUCTION

Healthcare utilisation (HCU) includes utilisation of hospital resources, home‐care resources, and physician resources and is important for healthcare planning (Sofianopoulou, Rushton, Rubin, & Pless‐Mulloli, 2012). A major component in HCU is health‐seeking behaviour (HSB), which is broadly defined as activities performed by individuals who perceive themselves as having a health problem or being ill, with the purpose of finding an appropriate remedy (Kasl & Cobb, 1966). Non‐optimal HSB may occur in several ways, such as refraining from seeking medical care entirely or not seeking care in accordance with expected needs. The conception of ‘need’ can be examined either objectively, using measures like poor health, the capacity to benefit from care, how much care a person requires to achieve health, or the care required to effect the maximum possible health improvement (Culyer & Wagstaff, 1993), or subjectively, using measures like ‘perception of need.’ HSB, like other factors such as socioeconomic circumstances and healthcare provider behaviour, is critical for access to healthcare services, and thus an important determinant of health (Braveman, Egerter, Cubbin, & Marchi, 2004). There are differences between socioeconomic groups in how people seek care. Socially vulnerable groups are less likely than non‐vulnerable groups to seek care in accordance with their expected needs (Agerholm, Bruce, Ponce de Leon, & Burstrom, 2013). Factors associated with HSB and HCU include level of income (Burstrom, 2002), gender (Westin, Ahs, Brand Persson, & Westerling, 2004), education level (Westin et al., 2004), perceived discrimination (Wamala, Merlo, Bostrom, & Hogstedt, 2007a), and lack of confidence in medical services (Westin et al., 2004).

Adherence is defined as the extent to which a person's behaviour, such as taking medication, following a diet, or executing lifestyle changes, corresponds with recommendations from a healthcare provider (WHO, 2003). Adherent behaviour to a medical treatment is often classified into primary and secondary adherence. Primary adherence refers to the patient filling a prescribed medication, and secondary adherence refers to the patient taking the medication as prescribed. Factors with a high probability of affecting adherence include demographics (Lowry, Dudley, Oddone, & Bosworth, 2005; Yiannakopoulou, Papadopulos, Cokkinos, & Mountokalakis, 2005), patient understanding and perception of medication (Berglund, Lytsy, & Westerling, 2012; WHO, 2003), sickness and treatment‐related factors (George, Kong, Thoman, & Stewart, 2005; Horne & Weinman, 1999; Leventhal & Cameron, 1987; Tordoff, Bagge, Gray, Campbell, & Norris, 2010), and locus of control (Berglund et al., 2012; Wilhelm, Rief, & Doering, 2018). Non‐adherence is a critical factor for treatment failure (Blackburn, Dobson, Blackburn, & Wilson, 2005; Cheng, Woo, Chan, Tomlinson, & You, 2004; Wei et al., 2002). Therefore, healthcare delivery and improving access to treatment are often considered public health concerns (Wamala, Merlo, Bostrom, Hogstedt, & Agren, 2007b). HSB is sometimes associated with adherence and shares similar risk factors in some cases (Fogarty et al., 2002; Samal et al., 2011). There is also a chronological relationship between HSB and adherence: to obtain prescription drugs, the patient must first seek care. Therefore, optimal healthcare is dependent on both HSB and adherence to medication utilisation. A critical factor to improve treatment outcomes is to identify key determinants of access to medication, HSB, and non‐adherence (Morris & Schulz, 1992).

Living environment and neighbourhood factors have been associated with health outcomes such as psychological distress, depression, well‐being, sleep quality, and overall health (Berglund, Westerling, & Lytsy, 2017; Hill, Burdette, & Hale, 2009; Hill, Ross, & Angel, 2005; Ou et al., 2018). The community socioeconomic context is seen as a contributor to poor health, independent of individual factors (Leventhal & Brooks‐Gunn, 2003; Ludwig et al., 2011; Robert, 1999; Sanbonmatsu et al., 2012). The local environment has also been associated with health‐related behaviours (De Greef, Van Dyck, Deforche, & De Bourdeaudhuij, 2011; Grinshteyn, Cunningham, Eisenman, Andersen, & Ettner, 2017; Karvonen & Rimpela, 1997; Sundquist, Malmstrom, & Johansson, 1999; Yen & Kaplan, 1998). Environmental factors, such as a disorderly neighbourhood, have been viewed as affecting individual health through a variety of mechanisms. These mechanisms include exposure to high levels of disorder, drug use, crime, social norms, poorer networks, lower social capital, and increased environmental stressors (Duncan, Duncan, & Strycker, 2002; Stockdale et al., 2007). Few studies have focused on environment and health behaviours such as HSB and adherence. However, it is possible that health behaviour also has associations with living context; certain factors in the living context may favour or disfavour HSB and adherence. An interesting consideration is how the living environment and social factors explain HSB and adherence to medication treatment. This study aims to investigate the associations between HSB for medical care and primary non‐adherent behaviour with housing type, outdoor neighbourhood safety, social support and informal caregiving in the general Swedish population.

## 2 METHODS

This study used data from the annual Swedish National Public Health Survey ‘Health on Equal Terms,’ carried out from 2004 to 2014 (Boström & Nyqvist, 2010). The National Public Health Survey is a repeated, cross‐sectional questionnaire study that has been distributed on a yearly basis since 2004 by Statistics Sweden on behalf of the Public Health Agency of Sweden (previously the Swedish National Institute of Public Health). The questionnaire contains roughly 85 questions. Each year, 20,000 people aged 16 to 84 years are randomly selected from the Swedish national population registry (from 2005 to 2007, 10,000 people were selected), adding up to a total of 190,000 persons for the period in this study. The questionnaires were returned by 100,433 individuals in total, making the response rate 52.9% (100,433/190,000). Analyses of non‐responders have been made through telephone interviews with non‐responders, but not every year. The main result from the analyses showed that non‐responders did not seem to have different response patterns than respondents in the telephone interviews (Boström, 2009).

### 2.1 Explanatory variables

The living environment was investigated with respect to two variables: housing type and behaviour based on perceived neighbourhood outdoor safety. Housing was categorised into three types: (a) private house; (b) condominium; and (c) rented apartment, lodger, dorm or other. The private house category included bungalows and townhouses, and the condominium category included apartments in housing cooperatives (*bostadsrätt*) and actual condominiums. Membership in a housing cooperative is generally considered the same thing as owning (as opposed to renting) an apartment and includes the formal right to use a specific apartment for an unlimited time, a right that can be bought and sold on the open real estate market. Housing cooperatives are the traditional form of owner‐occupied apartment housing in Sweden. The last category included living in various types of rental housing. Regarding the respondent's perception of their neighbourhood, a question about behaviour based on the perception of neighbourhood safety was used (Ferraro & Grange, 1987; Kullberg, Karlsson, Timpka, & Lindqvist, 2009). The question was phrased: ‘Do you ever refrain from going out alone for fear of being attacked, robbed or otherwise molested?’ Possible answers were ‘No,’ ‘Yes, sometimes,’ and ‘Yes, regularly.’ In this study, answers to the question were dichotomised into ‘Yes’ or ‘No.’ This question was not included in the questionnaire in 2004.

Social support is seen as an important factor for health outcomes (Rosell‐Murphy et al., 2014; Wheeler, Skinner, & Bailey, 2008), and has known associations with adherence (DiMatteo, 2004) (Lewandowski & Drotar, 2007). The questionnaire contained the following question regarding perceived emotional social support: ‘Do you have someone you can share your innermost feelings with and feel confident in?’ The following question was used to assess perceived instrumental social support: ‘Can you get help from someone/some people if you have practical problems or are ill?’ Answers to these two questions were dichotomised into either ‘yes’ or ‘no.’

Informal caregivers provide unpaid assistance and care to care recipients, most often an older or disabled family member, relative, friend, or neighbour. While caregiving may have several positive effects, it may also have deleterious effects on the caregiver's health‐related quality of life and can thus be considered a burden (Sjolander, Rolander, Jarhult, Martensson, & Ahlstrom, 2012). Studies have shown high risks of adverse physical and mental health, as well as institutionalisation and premature mortality among informal caregivers (Schulz, O'Brien, Bookwala, & Fleissner, 1995). The caregiving role may influence everyday life in several ways, as informal caregiving often involves extra work and responsibility. Since caregivers often focus time and attention on their care recipients, they may not prioritise their own health needs. Data on informal caregiving status were assessed with the following questions: ‘Have you an ill or old relative or friend whom you help with daily activities, see to or nurse?’ Answers to this question were dichotomised into ‘no’ or ‘yes.’

Demographic data used in this study were gender, age, and educational level (categorised as compulsory school, secondary school or equivalent, or university). Financial problems were assessed using the question: ‘During the last 12 months, have you had difficulties managing your current expenses for food, rent, bills, etc.?’ Answers to this question were dichotomised into ‘no’ or ‘yes.’

Information about long‐term illness was collected using the question: ‘Do you have any long‐term illness, problems following an accident, any disability, or any other long‐term health problem?’ Replies were phrased as ‘no’ or ‘yes.’ Background information on the questions in the Swedish National Public Health Survey has been published previously (Boström & Nyqvist, 2010).

### 2.2 Outcome variables

Refraining from seeking medical care was based on the question ‘During the past three months, have you considered yourself in need of medical care but refrained from seeking it?’ The response categories were ‘no’ or ‘yes.’ This question was not included in the questionnaire in 2005 or 2006.

Primary adherence was based on the question ‘Have you, during the past three months, refrained from buying medicine you were prescribed?’ The possible answers were ‘no’ or ‘yes.’ This question was not included in the questionnaire in 2007.

### 2.3 Analysis

Pearson's chi‐squared test and Student's *t*‐test were used to study differences between males and females as well as between the explanatory and outcome variables. Multiple binary logistic regressions were used to estimate associations between housing type, behaviour based on perceived outdoor neighbourhood safety, social support, caregiving status, demographics (gender, age, education level), financial problems, long‐term illness, and refraining from seeking care or primary non‐adherence. A stepwise approach was used with sets of explanatory variables to study associations with the outcome variables. Model 1 included housing type and neighbourhood safety. Model 2 included Model 1, social support and informal caregiving. Model 3 included Model 2, demographics and socioeconomics. Model 4 included Model 3 and long‐term illness. All tests were two‐sided, a *p*‐value ≤0.05 was considered statistically significant. Analyses of refraining from seeking medical care did not use data from 2005 or 2006, and analyses of adherence did not use data from 2007, due to the lack of outcome variables during these years. The Statistical Package for the Social Sciences (spss) version 20 (IBM spss Statistics for Windows, Armonk, NY: IBM Corp Chicago, IL, USA.) was used for the statistical analyses.

### 2.4 Ethical consideration

The Swedish National Public Health Survey was approved by the research ethics committee of the Swedish National Board of Health and Welfare (December 8, 2003). The present research study was approved by the regional ethical committee in Uppsala (May 10, 2016).

## 3 RESULTS

The study population was on average 50.7 years old (*SD* 18.0) and consisted of more women (56%) than men (44%). Compulsory school was the most common completed education level (45%). Private house was the most common housing type to live in (51%), followed by rented apartment, lodger, dorm or other (31%), and condominium (18%). A majority (77%) of the respondents reported not refraining from going out, but 23% did refrain from going outdoors due to perceived safety concerns. The distribution of variables in the study population is shown in Table 1.

**Table 1:** Distribution of characteristics among participants, by sex and for total group

		Male (N = 45,502)	Female (N = 54,931)	Total (N = 100,433)
Age	Mean (S.D.)	51.3 (17.9)**	50.2 (18.0)**	50.7 (18.0)
Education	Compulsory school	46.2**	43.7**	44.8
	Secondary school or equivalent	34.6**	32.6**	33.5
	University	19.2**	23.7**	21.7
Financial problems	No problems	87.6**	84.3**	85.8
	Have problems	12.4**	15.7**	14.2
Long‐term illness	No	62.9**	61.8**	62.3
	Yes	37.1**	38.2**	37.7
				
Housing type	Private house	53.6**	49.6**	51.4
	Condominium	16.7**	18.2**	17.5
	Rented apartment, lodger, dorm or other	29.7**	32.3**	31.1
Refraining from going out due to perceived low neighbourhood safety	No	91.0**	65.5**	77.1
	Yes	9.0**	34.5**	22.9
Emotional social support	Yes	87.1**	90.8**	89.1
	No	12.9**	9.2**	10.9
Instrumental social support	Yes	94.3**	95.5**	95.0
	No	5.7**	4.5**	5.0
Informal caregiver	No	90.2**	88.2**	89.1
	Yes	9.8**	11.8**	10.9
Refraining from seeking medical care	No	86.6**	83.4**	84.9
	Yes	13.4**	16.6**	15.1
Primary adherence to medication	No	94.3**	93.6**	93.9
	Yes	5.7**	6.4**	6.1

Figures as percentages if not stated otherwise. Pearson's chi‐squared test was used for distributions and Student's t‐test was used for age testing for differences between men and women.

*p ≤ 0.05, **p ≤ 0.01.

### 3.1 Refraining from seeking medical care

The majority of the responders reported not refraining from seeking medical care (85%). Refraining from seeking medical care was reported by 17% of females and 13% of males.

### 3.2 Adherence behaviour

In the total population, 6% showed primary non‐adherence to prescribed medication. Non‐adherence was reported by 6% of females and 6% of men.

### 3.3 Logistic regression models

Living in a rented apartment, lodger, dorm, or other was statistically significantly associated with refraining from seeking medical care (OR 1.78, 95% CI 1.71–1.85), and remained statistically significant in adjusted models (adjusted OR 1.16, 95% CI 1.00–1.22). Refraining from going out due to perception of an unsafe neighbourhood was associated with refraining from seeking medical care in an adjusted model (adjusted OR 1.59, 95% CI 1.51–1.67). Lower emotional social support was associated with refraining from seeking medical care (adjusted OR 1.77; 95% CI 1.66–1.90), and low instrumental social support was associated with refraining from seeking medical care (adjusted OR 1.79; 95% CI 1.63–1.97). Informal caregivers were more likely to refrain from seeking medical care (adjusted OR 1.46; 95% CI 1.37–1.57). Having problems with finances was associated with refraining from seeking medical care (adjusted OR 2.31; 95% CI 2.18–2.45). Having a long‐term illness was associated with a higher odds of refraining from seeking medical care (adjusted OR 2.61; 95% CI 2.49–2.74). See Table 2.

**Table 2:** Results of logistic regression models of factors explaining refraining from seeking medical care

		Crude OR 95% CI	Model 1 OR 95% CI	Model 2 OR 95% CI	Model 3 OR 95% CI	Model 4 OR 95% CI
Living environment	Housing type					
	Private house	1	1	1	1	1
	Condominium	1.18** (1.12 to 1.25)	1.13** (1.06 to 1.19)	1.08* (1.01 to 1.14)	1.06 (0.97 to 1.13)	1.05 (0.99 to 1.12)
	Rented apartment, lodger, dorm or other	1.78** (1.71 to 1.85)	1.67** (1.60 to 1.75)	1.55** (1.47 to 1.62)	1.20** (1.14 to 1.27)	1.16** (1.00 to 1.22)
	Refraining from going out due to perceived low neighbourhood safety					
	No	1	1	1	1	1
	Yes	1.94** (1.86 to 2.02)	1.85** (1.77 to 1.93)	1.79** (1.71 to 1.87)	1.65** (1.57 to 1.74)	1.59** (1.51 to 1.67)
Social support and informal caregiving	Emotional social support					
	Yes	1		1	1	1
	No	2.38** (2.26 to 2.50)		1.89** (1.77 to 2.01)	1.82** (1.70 to 1.94)	1.77** (1.66 to 1.90)
	Instrumental social support					
	Yes	1		1	1	1
	No	2.98** (2.79 to 3.19)		1.88** (1.73 to 2.05)	1.77** (1.62 to 1.94)	1.79** (1.63 to 1.97)
	Caregiver					
	No	1		1	1	1
	Yes	1.47** (1.39 to 1.55)		1.45** (1.36 to 1.55)	1.50** (1.40 to 1.60)	1.46** (1.37 to 1.57)
Demographic and socioeconomic	Gender					
	Male	1			1	1
	Female	1.29** (1.25 to 1.34)			1.13** (1.08 to 1.19)	1.12** (1.06 to 1.57)
	Age	0.99** (0.99 to 0.99)			0.99** (0.99 to 0.99)	0.98** (0.98 to 0.98)
	Education level					
	University	1			1	1
	Secondary school or equivalent	1.27** (1.20 to 1.33)			1.08* (1.02 to 1.15)	1.06 (0.99 to 1.13)
	Compulsory school	1.25** (1.19 to 1.32)			1.16** (1.09 to 1.23)	1.10** (1.04 to 1.17)
	Financial problems					
	No problems	1			1	1
	Have problems	3.32** (3.17 to 3.47)			2.60** (2.46 to 2.75)	2.31** (2.18 to 2.45)
Illness	Long‐term illness					
	No	1				1
	Yes	2.47** (2.37 to 2.56)				2.61** (2.49 to 2.74)

Odds ratio (OR), significance level, and confidence interval (CI) for having refrained from seeking medical care.

Model 1 = Housing type + Refraining from going out due to perceived low neighbourhood safety; Model 2 = Model 1 + Social support + informal caregiving; Model 3 = Demographic + financial problems; Model 4 = Model 3 + Long‐term illness.

*p ≤ 0.05, **p ≤ 0.01.

Living in a condominium had a statistically significant association with primary non‐adherence to medication in both a crude and an adjusted model (adjusted OR 1.13, 95% CI 1.03–1.23), see Table 3. Living in a rented apartment, lodger, dorm, or other was associated with non‐adherence in an adjusted regression model (adjusted OR 1.22; 95% CI 1.13–1.31). Refraining from going out due to the perception of an unsafe neighbourhood was associated with non‐adherence in an adjusted model (adjusted OR 1.26, 95% CI 1.17–1.36). Lower emotional social support was associated with non‐adherence (adjusted OR 1.30; 95% CI 1.18–1.42), and instrumental social support was also associated with non‐adherence (adjusted OR 1.79; 95% CI 1.59–2.01). Informal caregivers were more likely to be non‐adherent (adjusted OR 1.19; 95% CI: 1.08–1.31). Having problems with finances was associated with non‐adherence (adjusted OR 2.24; 95% CI 2.06–2.40). Having a long‐term illness was also associated with non‐adherence (adjusted OR 1.71; 95% CI 1.60–1.82).

**Table 3:** Results of logistic regression models of factors explaining primary non‐adherence to medication

		Crude OR 95% CI	Model 1 OR 95% CI	Model 2 OR 95% CI	Model 3 OR 95% CI	Model 4 OR 95% CI
Living environment	Housing type					
	Private house	1	1	1	1	1
	Condominium	1.26** (1.17 to 1.36)	1.19** (1.09 to 1.29)	1.13** (1.04 to 1.23)	1.14** (1.04 to 1.25)	1.13** (1.03 to 1.23)
	Rented apartment, lodger, dorm or other	1.71** (1.61 to 1.82)	1.61** (1.51 to 1.721)	1.46** (1.36 to 1.56)	1.25** (1.16 to 1.35)	1.22** (1.13 to 1.31)
	Refraining from going out due to perceived low neighbourhood safety					
	No	1	1	1	1	1
	Yes	1.52** (1.43 to 1.62)	1.46** (1.37 to 1.55)	1.39** (1.30 to 1.49)	1.30** (1.21 to 1.40)	1.26** (1.17 to 1.36)
Social support and informal caregiving	Emotional social support					
	Yes	1		1	1	1
	No	1.95** (1.81 to 2.09)		1.44** (1.32 to 1.57)	1.33** (1.21 to 1.46)	1.30** (1.18 to 1.42)
	Instrumental social support					
	Yes	1		1	1	1
	No	2.86** (2.62 to 3.12)		2.02** (1.80 to 2.26)	1.80** (1.60 to 2.03)	1.79** (1.59 to 2.01)
	Caregiver					
	No	1		1	1	1
	Yes	1.34** (1.23 to 1.45)		1.24** (1.13 to 1.35)	1.22** (1.11 to 1.34)	1.19** (1.08 to 1.31)
Demographic and socioeconomic	Gender					
	Male	1			1	1
	Female	1.13** (1.07 to 1.20)			1.05 (0.99 to 1.23)	1.04 (0.98 to 1.23)
	Age	0.996** (0.995 to 0.998)			1.00 (1.00 to 1.00)	0.996** (0.99 to 0.998)
	Education level					
	University	1			1	1
	Secondary school or equivalent	1.13** (1.05 to 1.23)			1.02 (0.93 to 1.11)	1.01 (0.92 to 1.10)
	Compulsory school	1.22** (1.13 to 1.31)			1.10* (1.01 to 1.20)	1.06 (0.98 to 1.16)
	Financial problems					
	No problems	1			1	1
	Have problems	2.97** (2.80 to 3.16)			2.39** (2.22 to 2.58)	2.23** (2.06 to 2.40)
Illness	Long‐term illness					
	No	1				1
	Yes	1.81** (1.71 to 1.91)				1.71** (1.60 to 1.82)

Odds ratio (OR), significance level, and confidence interval (CI) for primary non‐adherence to medication.

Model 1 = Housing type + Refraining from going out due to perceived low neighbourhood safety; Model 2 = Model 1 + Social support + informal caregiving; Model 3 = Demographic + financial problems; Model 4 = Model 3 + Long‐term illness.

*p ≤ 0.05, **p ≤ 0.01.

## 4 DISCUSSION

The aim of this study was to investigate whether attributes in the living environment and social factors were associated with HSB and adherence to prescribed medication. The main findings were that not living in a private house, refraining from going out in the neighbourhood because of perceived unsafety, and lacking social support or having the status of informal caregiver were separately associated with refraining from seeking medical care and non‐adherence to prescribed medication. Gender, age, education level, socioeconomic status and long‐term illness were further associated with HSB and adherence.

The local environment has been associated with health in several studies (Diez Roux, 2001). Fewer studies have investigated the associations between contextual factors and people's behaviour regarding seeking medical care and adherence to medication. Adherence behaviour to some treatments, such as those for type 2 diabetes mellitus and HIV, has been linked to environmental factors in some studies (de Vries McClintock et al., 2015; Surratt, Kurtz, Levi‐Minzi, & Chen, 2015). The results seen in this study may, to some extent, be related to housing and neighbourhoods as a marker for socioeconomic status (SES), since SES is known to have a large impact on health behaviour (Pampel, Krueger, & Denney, 2010). Contextual factors may also be considered mediators between SES and health, as SES may be linked to health through environmental exposures associated with different accommodations (Evans, 2004; Rauh, Landrigan, & Claudio, 2008). However, research also asserts that the local environment should not only be seen as a marker for SES (Hiscock, Macintyre, Kearns, & Ellaway, 2003); instead, the living environment may have a long‐term effect on residents’ health in several ways, such as variations in land use, building quality, design, transportation availability and infrastructure, access to nature/green/open space, public resources (e.g., services, healthcare, healthy food, schools, recreational opportunities), street condition, cleanliness/garbage maintenance, traffic volume, air quality, and noise (Corburn, 2005; Rollings, Wells, & Evans, 2015; World Health Organization and UN‐Habitat, 2016).

The results show that HSB and adherence were associated with refraining from going out due to a perceived unsafe neighbourhood; these results are in line with previous research that identified the perceptions of crime and violence as predictors of health (Stockdale et al., 2007). Fear of crime and safety‐related worries are suggested to have an impact on behaviour, potentially decreasing physical activity (Gomez, Johnson, Selva, & Sallis, 2004) and limiting personal freedom (Kullberg et al., 2009). A previous study of safety‐related aspects found that factors typically associated with safety were associated with physical activity among children and adolescents (Carver, Timperio, Hesketh, & Crawford, 2010). Also, the perception of an unsafe environment may have an impact on people's healthcare use, which the results in this study imply. Safety‐related worry might lead to ‘time‐space inequalities,’ which is a theory arguing that the ability to access and utilise different spaces during certain times varies with subgroups, and has a disproportionate negative impact on certain subgroups, for example, low‐income mothers (Whitley & Prince, 2005). Such inequalities have been found to have implications for mental health (Grinshteyn et al., 2017; Whitley & Prince, 2005) and physical activity (Bennett et al., 2007), and might also have implications for access to care and adherence to medication. The results in this study imply that the healthcare system in Sweden may not be able to fully balance out health inequalities between people in living in different environments, as HSB and adherence may also vary due to environmental factors.

Social support is, to a certain extent, also a contextual factor (Williams, Barclay, & Schmied, 2004) (Ariza‐Montes, Leal‐Rodriguez, Rodriguez‐Felix, & Albort‐Morant, 2017), and previous research have found that some aspects of social capital, such as trust in the community, are slightly associated with refraining from medical care in smaller communities (Mizuochi, 2016). In theoretical models, social support and potential health outcomes, health behaviour is included as an underlying link between support and health (Uchino, 2006; Umberson, 1987). The hypothesis is that social support is health‐promoting because it facilitates healthier behaviours in two major ways, either directly (e.g., practical support) or indirectly (e.g., social support impacts the meaning of life). In this study, lower social support was associated with refraining from seeking medical care and non‐adherence, as was seen in previous studies regarding adherence (DiMatteo, 2004; Gomes‐Villas Boas, Foss, Freitas, & Pace, 2012).

It is reasonable to assume that caregivers, who often provide assistance with medical concerns to care receivers, would be more familiar both with the healthcare system and the importance of adherence to prescribed medications, thus simplifying their own health seeking and adherence to treatment. However, the findings in this study suggest that the caregiving role was associated with less utilisation of medical care and prescribed medication for caregivers' own healthcare needs. These findings are consistent with previous studies showing that the excess of physical and psychological health problems among caregivers does not lead to an expected higher level of medical care utilisation (Baumgarten et al., 1997), and that informal caregivers often have unmet care needs (Wang, Molassiotis, Chung, & Tan, 2018). Informal caregiving is a resource in several ways, for instance as an adherence‐enhancing factor for care receivers (Trivedi, Bryson, Udris, & Au, 2012).

In Sweden, there are some publically financed benefits for supporting caregivers. When a relative is severely ill, with a potentially life threatening condition, it is possible for an employed caregiver to receive reimbursement through social health insurance for a shorter period of time. Municipalities must also, under national law, provide support to close relatives of individuals with mental and physical diseases or disabilities, as well as to persons with addiction or dependence problems. Possible support includes information, education, support and relief in caregiving and financial support. When the National Board of Health and Welfare evaluated this policy in 2014, it was concluded that user organisations were critical of the lack of support and differences in quality of support given in different regions (National Board of Health and Welfare in Sweden, 2014). Another critique was that while there are regulations for caregiving support within social services, there is no equivalent within the regulations of health services, undermining overall support to caregivers. The Swedish universal healthcare system has been criticised for its difficulties in addressing informal caregivers (Johansson, Long, & Parker, 2011). Also, one study of informal caregivers to dementia care receivers showed that informal caregivers in Sweden spend less time in their caregiving when compared with caregivers in another European country, Italy (Chiatti et al., 2018). Systems for supporting informal caregivers are present in other European countries as well, such as the United Kingdom's Carer's Assessments for adult carers of people who are disabled, ill or elderly and the Carer Support Needs Assessment Tool (Austin, Ewing, & Grande, 2017). However, caregivers perform a variety of tasks that differ between families, which may complicate the policy‐making and design of a general intervention for this group. Some interventions may influence regarding caregivers' health, such as health communication, development of problem‐solving skills, information and communication technologies (Frambes, Given, Lehto, Sikorskii, & Wyatt, 2018; Lucero et al., 2018).

Nevertheless, caregivers do not seem to support their own adherence to prescribed medication to a greater extent, as they reported lower primary adherence than the general population. These results are similar to those of previous studies which show that caregiving status is associated with secondary non‐adherence (Shrank et al., 2011). Caregivers are in an unusual situation as they may have unmet health needs themselves, but they are at the same time a part of the healthcare chain as informal providers. It could be advantageous to implement a new caring model which includes the caregiver's own needs (not only his/her knowledge and skills) as a part of patient care. Such a caregiver‐focused model could capture the caregiver's needs along with the care receiver's. This model could be based on three major constructs in addition to formal care: the care receiver's medical needs, health needs that are provided for by an informal caregiver, and the caregiver's own long‐term needs, required for him/her to function as a caregiver. If these three aspects could be equally considered, supported, and balanced, it would result in a sustainable and healthy caring situation for the patient‐caregiver dyad. Such a model also takes advantage of the position within the healthcare chain that many caregivers have, and this approach could help caregivers seek the care they need.

This study demonstrated that several indicators of vulnerability, such as low social support, SES, and long‐term illness were associated with refraining from seeking medical care and medical non‐adherence. Similar results have been seen in previous studies regarding factors related to vulnerable groups (Agerholm et al., 2013; Burstrom, 2002; Westin et al., 2004). However, after adjusting for factors associated with vulnerable groups, such as low education level and financial problems, the associations between living environment, social support and caregiving remained statistically significant.

Healthcare utilisation has been linked to health outcomes (Williams, Eckert, L'Italien, Lapuerta, & Weinberger, 2003), and HSB is considered to be a pathway through which other risk factors (such as socioeconomic status) can influence health outcomes (Benova, Grundy, & Ploubidis, 2015). Most people in this study did not refrain from seeking care and did not report insufficient primary adherence to medical treatment. However, from a perspective of community health and spatial policy, the differences due to local environment, social support, and caregiving status are of considerable importance. The results highlight the importance of factors in the living environment for health behaviour, and show that the healthcare system (which has the potential to balance out differences in health outcome) needs to be focused on context‐based interventions.

### 4.1 Strengths and limitations

The main strengths of this study are the large sample size and the simple random sampling of the study population, which make the results broadly generalisable. The main limitation of the study is that outcomes were self‐reported, and it is uncertain to what extent reported health behaviour corresponds to actual behaviour. The study sample excludes people of 85 years of age or older, and the results should not be generalised beyond the age of the study population. There was no information about how ill‐health affected the responses, but it is reasonable to assume that it decreased the capability to respond. Since having long‐term illness was a factor associated with both refraining from seeking medical care and being non‐adherent, it is likely that the present sample underestimates the impact of having chronic disease on the outcomes. Moreover, the data were cross‐sectional, and it is not possible to determine the cause‐effect relationship between predictor variables and outcomes. More research needs to be carried out to investigate the mechanisms of the observed differences in HSB and adherence and which factors mediate the associations between predictors and outcomes. There is a possibility that we have not included some important confounders, such as ethnicity. Furthermore, there is a risk of selection bias in all studies using self‐reported questionnaire data.

## 5 CONCLUSION

This study suggests that living in rental housing, refraining from going out due to neighbourhood safety concerns, lack of social support or informal caregiver status are associated with lower HSB and non‐adherence to prescribed medication. These results were independent of demographics, socioeconomic status, and long‐term illness. The mechanisms of these associations need to be further investigated in different types of studies of context, health behaviour, and processes of healthcare delivery. As a suggestion, qualitative research in specific patient groups may be beneficial for detailing information. In addition, experimental research is needed to investigate the impact of different interventions and changes in policy.

## CONFLICTS OF INTEREST

None declared.

## References

[hsc12758-bib-0001] Agerholm, J. , Bruce, D. , Ponce de Leon, A. , & Burstrom, B. (2013). Socioeconomic differences in healthcare utilization, with and without adjustment for need: An example from Stockholm, Sweden. Scandinavian Journal of Public Health, 41(3), 318–325. 10.1177/1403494812473205 23406653

[hsc12758-bib-0002] Ariza‐Montes, A. , Leal‐Rodriguez, A. L. , Rodriguez‐Felix, L. , & Albort‐Morant, G. (2017). Can an internal locus of control and social support reduce work‐related levels of stress and strain?: A comparative study between spanish owners and managers. Journal of Occupational and Environmental Medicine, 59(9), 903–912. 10.1097/jom.0000000000001096 28692007

[hsc12758-bib-0003] Austin, L. , Ewing, G. , & Grande, G. (2017). Factors influencing practitioner adoption of carer‐led assessment in palliative homecare: A qualitative study of the use of the Carer Support Needs Assessment Tool (CSNAT). PLoS ONE, 12(6), e0179287 10.1371/journal.pone.0179287 28622348 PMC5473540

[hsc12758-bib-0004] Baumgarten, M. , Battista, R. N. , Infante‐Rivard, C. , Hanley, J. A. , Becker, R. , Bilker, W. B. , & Gauthier, S. (1997). Use of physician services among family caregivers of elderly persons with dementia. Journal of Clinical Epidemiology, 50(11), 1265–1272. 10.1016/S0895-4356(97)00168-6 9393382

[hsc12758-bib-0005] Bennett, G. G. , McNeill, L. H. , Wolin, K. Y. , Duncan, D. T. , Puleo, E. , & Emmons, K. M. (2007). Safe to walk? Neighborhood safety and physical activity among public housing residents. PLoS Medicine, 4(10), e306 10.1371/journal.pmed.0040306 PMC2039759 17958465

[hsc12758-bib-0006] Benova, L. , Grundy, E. , & Ploubidis, G. B. (2015). Socioeconomic position and health‐seeking behavior for hearing loss among older adults in England. The Journals of Gerontology Series B: Psychological Sciences and Social Sciences, 70(3), 443–452. 10.1093/geronb/gbu024 PMC4501830 24663332

[hsc12758-bib-0007] Berglund, E. , Lytsy, P. , & Westerling, R. (2012). Adherence to and beliefs in lipid‐lowering medical treatments: A structural equation modeling approach including the necessity‐concern framework. Patient Education and Counseling, 91, 105–112. 10.1016/j.pec.2012.11.001 23218590

[hsc12758-bib-0008] Berglund, E. , Westerling, R. , & Lytsy, P. (2017). Housing type and neighbourhood safety behaviour predicts self‐rated health, psychological well‐being and frequency of recent unhealthy days: A comparative cross‐sectional study of the general population in Sweden. Planning Practice & Research, 32(4), 444–465. 10.1080/02697459.2017.1374706

[hsc12758-bib-0009] Blackburn, D. F. , Dobson, R. T. , Blackburn, J. L. , & Wilson, T. W. (2005). Cardiovascular morbidity associated with nonadherence to statin therapy. Pharmacotherapy, 25(8), 1035–1043. 10.1592/phco.2005.25.8.1035 16207093

[hsc12758-bib-0010] Boström, G. (2009). Vad betyder bortfallet för resultatet i folkhälsoenkäter? [What does the loss of the result in public health surveys mean?]. Retrieved from https://www.folkhalsomyndigheten.se/contentassets/ae5c19f5eb6d44bd81411de62530a04c/nationella-folkhalsoenkaten-vad_betyder_bortfallet-100330.pdf (accessed 22 October 2018).

[hsc12758-bib-0011] Boström, G. , & Nyqvist, K. (2010). Objective and background of the questions in the national public health survey. Retrieved from Östersund: http://www.folkhalsomyndigheten.se/publicerat-material/publikationer/Objective-and-background-of-the-questions-in-the-national-public-health-survey/ (accessed 22 October 2018).

[hsc12758-bib-0012] Braveman, P. A. , Egerter, S. A. , Cubbin, C. , & Marchi, K. S. (2004). An approach to studying social disparities in health and health care. American Journal of Public Health, 94(12), 2139–2148. 10.2105/AJPH.94.12.2139 15569966 PMC1448604

[hsc12758-bib-0013] Burstrom, B. (2002). Increasing inequalities in health care utilisation across income groups in Sweden during the 1990s? Health Policy, 62(2), 117–129. 10.1016/s0168-8510(02)00016-7 12354407

[hsc12758-bib-0014] Carver, A. , Timperio, A. , Hesketh, K. , & Crawford, D. (2010). Are safety‐related features of the road environment associated with smaller declines in physical activity among youth? Journal of Urban Health, 87(1), 29–43. 10.1007/s11524-009-9402-3 19949995 PMC2821603

[hsc12758-bib-0015] Cheng, C. W. , Woo, K. S. , Chan, J. C. , Tomlinson, B. , & You, J. H. (2004). Association between adherence to statin therapy and lipid control in Hong Kong Chinese patients at high risk of coronary heart disease. British Journal of Clinical Pharmacology, 58(5), 528–535. 10.1111/j.1365-2125.2004.02202.x 15521901 PMC1884622

[hsc12758-bib-0016] Chiatti, C. , Rodriguez Gatta, D. , Malmgren Fange, A. , Scandali, V. M. , Masera, F. , & Lethin, C. (2018). Utilization of formal and informal care by community‐living people with dementia: A comparative study between Sweden and Italy. International Journal of Environmental Research and Public Health, 15(12), 2679 10.3390/ijerph15122679 PMC6313614 30487417

[hsc12758-bib-0017] Corburn, J. (2005). Urban planning and health disparities: Implications for research and practice. Planning Practice & Research, 20(2), 111–126. 10.1080/02697450500414652

[hsc12758-bib-0018] Culyer, A. J. , & Wagstaff, A. (1993). Equity and equality in health and health care. Journal of Health Economics, 12(4), 431–457. 10.1016/0167-6296(93)90004-X 10131755

[hsc12758-bib-0019] De Greef, K. , Van Dyck, D. , Deforche, B. , & De Bourdeaudhuij, I. (2011). Physical environmental correlates of self‐reported and objectively assessed physical activity in Belgian type 2 diabetes patients. Health & Social Care in the Community, 19(2), 178–188. 10.1111/j.1365-2524.2010.00958.x 20880106

[hsc12758-bib-0020] de Vries McClintock, H. F. , Wiebe, D. J. , O’Donnell, A. J. , Morales, K. H. , Small, D. S. , & Bogner, H. R. (2015). Neighborhood social environment and patterns of adherence to oral hypoglycemic agents among patients with type 2 diabetes mellitus. Family & Community Health, 38(2), 169–179. 10.1097/FCH.0000000000000069 25739064 PMC4351782

[hsc12758-bib-0021] Diez Roux, A. V. (2001). Investigating neighborhood and area effects on health. American Journal of Public Health, 91(11), 1783–1789. 10.2105/AJPH.91.11.1783 11684601 PMC1446876

[hsc12758-bib-0022] DiMatteo, M. R. (2004). Social support and patient adherence to medical treatment: A meta‐analysis. Health Psychology, 23(2), 207–218. 10.1037/0278-6133.23.2.207 15008666

[hsc12758-bib-0023] Duncan, S. C. , Duncan, T. E. , & Strycker, L. A. (2002). A multilevel analysis of neighborhood context and youth alcohol and drug problems. Prevention Science, 3(2), 125–133. 12088137 10.1023/a:1015483317310

[hsc12758-bib-0024] Evans, G. W. (2004). The environment of childhood poverty. American Psychologist, 59(2), 77–92. 10.1037/0003-066x.59.2.77 14992634

[hsc12758-bib-0025] Ferraro, K. F. , & Grange, R. L. (1987). The measurement of fear of crime*. Sociological Inquiry, 57(1), 70–97. 10.1111/j.1475-682X.1987.tb01181.x

[hsc12758-bib-0026] Fogarty, L. , Roter, D. , Larson, S. , Burke, J. , Gillespie, J. , & Levy, R. (2002). Patient adherence to HIV medication regimens: A review of published and abstract reports. Patient Education and Counseling, 46(2), 93–108. 10.1016/S0738-3991(01)00219-1 11867239

[hsc12758-bib-0027] Frambes, D. , Given, B. , Lehto, R. , Sikorskii, A. , & Wyatt, G. (2018). Informal caregivers of cancer patients: Review of interventions, care activities, and outcomes. Western Journal of Nursing Research, 40(7), 1069–1097. 10.1177/0193945917699364 28381113

[hsc12758-bib-0028] George, J. , Kong, D. C. , Thoman, R. , & Stewart, K. (2005). Factors associated with medication nonadherence in patients with COPD. Chest, 128(5), 3198–3204. 10.1378/chest.128.5.3198 16304262

[hsc12758-bib-0029] Gomes‐Villas Boas, L. C. , Foss, M. C. , Freitas, M. C. , & Pace, A. E. (2012). Relationship among social support, treatment adherence and metabolic control of diabetes mellitus patients. Revista Latino‐Americana de Enfermagem, 20(1), 52–58. 10.1590/S0104-11692012000100008 22481721

[hsc12758-bib-0030] Gomez, J. E. , Johnson, B. A. , Selva, M. , & Sallis, J. F. (2004). Violent crime and outdoor physical activity among inner‐city youth. Preventive Medicine, 39(5), 876–881. 10.1016/j.ypmed.2004.03.019 15475019

[hsc12758-bib-0031] Grinshteyn, E. G. , Cunningham, W. E. , Eisenman, D. P. , Andersen, R. , & Ettner, S. L. (2017). Fear of violent crime and anxiety/depression among adolescents. Mental Health and Prevention, 8, 39–45. 10.1016/j.mhp.2017.07.002

[hsc12758-bib-0032] Hill, T. D. , Burdette, A. M. , & Hale, L. (2009). Neighborhood disorder, sleep quality, and psychological distress: Testing a model of structural amplification. Health & Place, 15(4), 1006–1013. 10.1016/j.healthplace.2009.04.001 19447667

[hsc12758-bib-0033] Hill, T. D. , Ross, C. E. , & Angel, R. J. (2005). Neighborhood disorder, psychophysiological distress, and health. Journal of Health and Social Behavior, 46(2), 170–186. 10.1177/002214650504600204 16028456

[hsc12758-bib-0034] Hiscock, R. , Macintyre, S. , Kearns, A. , & Ellaway, A. (2003). Residents and residence: Factors predicting the health disadvantage of social renters compared to owner‐occupiers. Journal of Social Issues, 59(3), 527–546. 10.1111/1540-4560.00076

[hsc12758-bib-0035] Horne, R. , & Weinman, J. (1999). Patients’ beliefs about prescribed medicines and their role in adherence to treatment in chronic physical illness. Journal of Psychosomatic Research, 47(6), 555–567. 10.1016/S0022-3999(99)00057-4 10661603

[hsc12758-bib-0036] Johansson, L. , Long, H. , & Parker, M. G. (2011). Informal caregiving for elders in Sweden: An analysis of current policy developments. Journal of Aging & Social Policy, 23(4), 335–353. 10.1080/08959420.2011.605630 21985063

[hsc12758-bib-0037] Karvonen, S. , & Rimpela, A. H. (1997). Urban small area variation in adolescents’ health behaviour. Social Science & Medicine, 45(7), 1089–1098. 10.1016/S0277-9536(97)00036-1 9257400

[hsc12758-bib-0038] Kasl, S. V. , & Cobb, S. (1966). Health behavior, illness behavior, and sick role behavior. I. Health and illness behavior. Archives of Environmental Health, 12(2), 246–266. 10.1080/00039896.1966.10664365 5322534

[hsc12758-bib-0039] Kullberg, A. , Karlsson, N. , Timpka, T. , & Lindqvist, K. (2009). Correlates of local safety‐related concerns in a Swedish Community: A cross‐sectional study. BMC Public Health, 9, 221 10.1186/1471-2458-9-221 19586534 PMC2724512

[hsc12758-bib-0040] Leventhal, H. , & Cameron, L. (1987). Behavioral theories and the problem of compliance. Patient Education and Counseling, 10(2), 117–138. 10.1016/0738-3991(87)90093-0

[hsc12758-bib-0041] Leventhal, T. , & Brooks‐Gunn, J. (2003). Moving to opportunity: An experimental study of neighborhood effects on mental health. American Journal of Public Health, 93(9), 1576–1582. 10.2105/AJPH.93.9.1576 12948983 PMC1448013

[hsc12758-bib-0042] Lewandowski, A. , & Drotar, D. (2007). The relationship between parent‐reported social support and adherence to medical treatment in families of adolescents with type 1 diabetes. Journal of Pediatric Psychology, 32(4), 427–436. 10.1093/jpepsy/jsl037 17056638

[hsc12758-bib-0043] Lowry, K. P. , Dudley, T. K. , Oddone, E. Z. , & Bosworth, H. B. (2005). Intentional and unintentional nonadherence to antihypertensive medication. The Annals of Pharmacotherapy, 39(7–8), 1198–1203. 10.1345/aph.1E594 15956238

[hsc12758-bib-0044] Lucero, R. J. , Fehlberg, E. A. , Patel, A. G. M. , Bjarnardottir, R. I. , Williams, R. , Lee, K. , … Mittelman, M. (2018). The effects of information and communication technologies on informal caregivers of persons living with dementia: A systematic review. Alzheimer's & Dementia: Translational Research & Clinical Interventions, 5, 1–12. 10.1016/j.trci.2018.11.003 30623020 PMC6315277

[hsc12758-bib-0045] Ludwig, J. , Sanbonmatsu, L. , Gennetian, L. , Adam, E. , Duncan, G. J. , Katz, L. F. , … McDade, T. W. (2011). Neighborhoods, obesity, and diabetes—A randomized social experiment. New England Journal of Medicine, 365(16), 1509–1519. 10.1056/NEJMsa1103216 22010917 PMC3410541

[hsc12758-bib-0046] Mizuochi, M. (2016). Social capital and refraining from medical care among elderly people in Japan. BMC Health Services Research, 16, 331–331. 10.1186/s12913-016-1599-8 27484252 PMC4970323

[hsc12758-bib-0047] Morris, L. S. , & Schulz, R. M. (1992). Patient compliance–an overview. Journal of Clinical Pharmacy and Therapeutics, 17(5), 283–295. 10.1111/j.1365-2710.1992.tb01306.x 1464632

[hsc12758-bib-0048] National Board of Health and Welfare in Sweden . (2014). Socialstyrelsen. Stöd till personer som vårdar och stödjer närstående. Slutrapport 2014, [Support for people who care and support relatives. Final report 2014]. Stockholm, Sweden: Socialstyrelsen.

[hsc12758-bib-0049] Ou, J. Y. , Peters, J. L. , Levy, J. I. , Bongiovanni, R. , Rossini, A. , & Scammell, M. K. (2018). Self‐rated health and its association with perceived environmental hazards, the social environment, and cultural stressors in an environmental justice population. BMC Public Health, 18(1), 970–970. 10.1186/s12889-018-5797-7 30075713 PMC6090753

[hsc12758-bib-0050] Pampel, F. C. , Krueger, P. M. , & Denney, J. T. (2010). Socioeconomic Disparities in Health Behaviors. Annual Review of Sociology, 36, 349–370. 10.1146/annurev.soc.012809.102529 PMC3169799 21909182

[hsc12758-bib-0051] Rauh, V. A. , Landrigan, P. J. , & Claudio, L. (2008). Housing and health: Intersection of poverty and environmental exposures. Annals of the New York Academy of Sciences, 1136, 276–288. 10.1196/annals.1425.032 18579887

[hsc12758-bib-0052] Robert, S. A. (1999). SOCIOECONOMIC POSITION AND HEALTH: The independent contribution of community socioeconomic context. Annual Review of Sociology, 25(1), 489–516. 10.1146/annurev.soc.25.1.489

[hsc12758-bib-0053] Rollings, K. A. , Wells, N. M. , & Evans, G. W. (2015). Measuring physical neighborhood quality related to health. Behavioral Sciences, 5(2), 190–202. 10.3390/bs5020190 25938692 PMC4493443

[hsc12758-bib-0054] Rosell‐Murphy, M. , Bonet‐Simó, J. M. , Baena, E. , Prieto, G. , Bellerino, E. , Solé, F. , … Mimoso, S. (2014). Intervention to improve social and family support for caregivers of dependent patients: ICIAS study protocol. BMC Family Practice, 15, 53 10.1186/1471-2296-15-53 24666438 PMC4230240

[hsc12758-bib-0055] Samal, L. , Saha, S. , Chander, G. , Korthuis, P. T. , Sharma, R. K. , Sharp, V. , … Beach, M. C. (2011). Internet health information seeking behavior and antiretroviral adherence in persons living with HIV/AIDS. AIDS Patient Care STDS, 25(7), 445–449. 10.1089/apc.2011.0027 21682586 PMC3159122

[hsc12758-bib-0056] Sanbonmatsu, L. , Potter, N. A. , Adam, E. , Duncan, G. J. , Katz, L. F. , Kessler, R. C. ,… McDade, T. W. (2012). The long‐term effects of moving to opportunity on adult health and economic self‐sufficiency. Cityscape, 14(2), 109–136.

[hsc12758-bib-0057] Schulz, R. , O’Brien, A. T. , Bookwala, J. , & Fleissner, K. (1995). Psychiatric and physical morbidity effects of dementia caregiving: Prevalence, correlates, and causes. The Gerontologist, 35(6), 771–791. 10.1093/geront/35.6.771 8557205

[hsc12758-bib-0058] Shrank, W. H. , Liberman, J. N. , Fischer, M. A. , Kilabuk, E. , Girdish, C. , Cutrona, S. , … Choudhry, N. K. (2011). Are caregivers adherent to their own medications? Journal of the American Pharmacists Association, 51(4), 492–498. 10.1331/JAPhA.2011.10006 21602166

[hsc12758-bib-0059] Sjolander, C. , Rolander, B. , Jarhult, J. , Martensson, J. , & Ahlstrom, G. (2012). Health‐related quality of life in family members of patients with an advanced cancer diagnosis: A one‐year prospective study. Health and Quality of Life Outcomes, 10, 89 10.1186/1477-7525-10-89 22846452 PMC3489687

[hsc12758-bib-0060] Sofianopoulou, E. , Rushton, S. , Rubin, G. , & Pless‐Mulloli, T. (2012). Defining GP practice areas based on true service utilisation. Health Place, 18(6), 1248–1254. 10.1016/j.healthplace.2012.08.006 23041911

[hsc12758-bib-0061] Stockdale, S. E. , Wells, K. B. , Tang, L. , Belin, T. R. , Zhang, L. , & Sherbourne, C. D. (2007). The importance of social context: Neighborhood stressors, stress‐buffering mechanisms, and alcohol, drug, and mental health disorders. Social Science & Medicine, 65(9), 1867–1881. 10.1016/j.socscimed.2007.05.045 17614176 PMC2151971

[hsc12758-bib-0062] Sundquist, J. , Malmstrom, M. , & Johansson, S. E. (1999). Cardiovascular risk factors and the neighbourhood environment: A multilevel analysis. International Journal of Epidemiology, 28(5), 841–845. 10.1093/ije/28.5.841 10597980

[hsc12758-bib-0063] Surratt, H. L. , Kurtz, S. P. , Levi‐Minzi, M. A. , & Chen, M. (2015). Environmental influences on HIV medication adherence: The role of neighborhood disorder. American Journal of Public Health, 105(8), 1660–1666. 10.2105/AJPH.2015.302612 26066966 PMC4498986

[hsc12758-bib-0064] Tordoff, J. M. , Bagge, M. L. , Gray, A. R. , Campbell, A. J. , & Norris, P. T. (2010). Medicine‐taking practices in community‐dwelling people aged > or =75 years in New Zealand. Age and Ageing, 39(5), 574–580. 10.1093/ageing/afq069 20558482

[hsc12758-bib-0065] Trivedi, R. B. , Bryson, C. L. , Udris, E. , & Au, D. H. (2012). The influence of informal caregivers on adherence in COPD patients. Annals of Behavioral Medicine, 44(1), 66–72. 10.1007/s12160-012-9355-8 22422104

[hsc12758-bib-0066] Uchino, B. N. (2006). Social support and health: A review of physiological processes potentially underlying links to disease outcomes. Journal of Behavioral Medicine, 29(4), 377–387. 10.1007/s10865-006-9056-5 16758315

[hsc12758-bib-0067] Umberson, D. (1987). Family status and health behaviors: Social control as a dimension of social integration. Journal of Health and Social Behavior, 28(3), 306–319. 10.2307/2136848 3680922

[hsc12758-bib-0068] Wamala, S. , Merlo, J. , Bostrom, G. , & Hogstedt, C. (2007a). Perceived discrimination, socioeconomic disadvantage and refraining from seeking medical treatment in Sweden. Journal of Epidemiology and Community Health, 61(5), 409–415. 10.1136/jech.2006.049999 17435207 PMC2465685

[hsc12758-bib-0069] Wamala, S. , Merlo, J. , Bostrom, G. , Hogstedt, C. , & Agren, G. (2007b). Socioeconomic disadvantage and primary non‐adherence with medication in Sweden. International Journal for Quality in Health Care, 19(3), 134–140. 10.1093/intqhc/mzm011 17449480

[hsc12758-bib-0070] Wang, T. , Molassiotis, A. , Chung, B. P. M. , & Tan, J.‐Y. (2018). Unmet care needs of advanced cancer patients and their informal caregivers: A systematic review. BMC Palliative Care, 17(1), 96–96. 10.1186/s12904-018-0346-9 30037346 PMC6057056

[hsc12758-bib-0071] Wei, L. , Wang, J. , Thompson, P. , Wong, S. , Struthers, A. D. , & MacDonald, T. M. (2002). Adherence to statin treatment and readmission of patients after myocardial infarction: A six year follow up study. Heart, 88(3), 229–233. 10.1136/heart.88.3.229 12181210 PMC1767352

[hsc12758-bib-0072] Westin, M. , Ahs, A. , Brand Persson, K. , & Westerling, R. (2004). A large proportion of Swedish citizens refrain from seeking medical care–lack of confidence in the medical services a plausible explanation? Health Policy, 68(3), 333–344. 10.1016/j.healthpol.2003.10.008 15113644

[hsc12758-bib-0073] Wheeler, A. C. , Skinner, D. G. , & Bailey, D. B. (2008). Perceived quality of life in mothers of children with fragile X syndrome. American Journal of Mental Retardation, 113(3), 159–177. 10.1352/0895-8017(2008)113[159:PQOLIM]2.0.CO;2 18407719

[hsc12758-bib-0074] Whitley, R. , & Prince, M. (2005). Fear of crime, mobility and mental health in inner‐city London, UK. Social Science & Medicine, 61(8), 1678–1688. 10.1016/j.socscimed.2005.03.044 15908089

[hsc12758-bib-0075] WHO . (2003). Adherence to long‐term therapies: Evidence for action. Geneva, Switzerland: World Health Organization.

[hsc12758-bib-0076] World Health Organization and UN-Habitat . (2016). Global report on urban health: Equitable healthier cities for sustainable development. World Health Organization.

[hsc12758-bib-0077] Wilhelm, M. , Rief, W. , & Doering, B. K. (2018). It’s all a matter of necessity and concern: A structural equation model of adherence to antihypertensive medication. Patient Education and Counseling, 101(3), 497–503. 10.1016/j.pec.2017.09.007 28964558

[hsc12758-bib-0078] Williams, L. S. , Eckert, G. J. , L’Italien, G. J. , Lapuerta, P. , & Weinberger, M. (2003). Regional variation in health care utilization and outcomes in ischemic stroke. Journal of Stroke and Cerebrovascular Diseases, 12(6), 259–265. 10.1016/j.jstrokecerebrovasdis.2003.09.008 17903937

[hsc12758-bib-0079] Williams, P. , Barclay, L. , & Schmied, V. (2004). Defining social support in context: A necessary step in improving research, intervention, and practice. Qualitative Health Research, 14(7), 942–960. 10.1177/1049732304266997 15296665

[hsc12758-bib-0080] Yen, I. H. , & Kaplan, G. A. (1998). Poverty area residence and changes in physical activity level: Evidence from the Alameda County Study. American Journal of Public Health, 88(11), 1709–1712. 10.2105/AJPH.88.11.1709 9807543 PMC1508581

[hsc12758-bib-0081] Yiannakopoulou, E. , Papadopulos, J. S. , Cokkinos, D. V. , & Mountokalakis, T. D. (2005). Adherence to antihypertensive treatment: A critical factor for blood pressure control. European Journal of Cardiovascular Prevention and Rehabilitation, 12(3), 243–249. 10.1097/00149831-200506000-00010 15942423

